# Supplementary material for: Effectiveness and Safety of Ayurvedic Medicines in Type 2 Diabetes Mellitus Management: A Systematic Review and Meta-Analysis
Source: Front Pharmacol. 2022 Jun 8;13:821810. doi: 10.3389/fphar.2022.821810 (PMC9213670; doi:10.3389/fphar.2022.821810)
Supplement: Supplementary file 1 [file DataSheet1.zip › Supplementary Material/Forest and Funnel Plots/Gynostemma pentaphyllum/PPBG.pdf]

Gynostemma pentaphyllum - PPBG

Study	Intervention N	Intervention Mean	Intervention SD	Comparator N	Comparator Mean	Comparator SD	Mean Diff. with 95% CI	Weight (%)
Huyen 2010	12	-63.06	102.69	12	1.80	124.31	-64.86 [-156.09, 26.37]	35.65
Huyen 2012	12	-64.86	113.50	13	23.42	50.44	-88.28 [-156.18, -20.38]	64.35
**Overall**							-79.93 [-134.40, -25.46]	

Heterogeneity: $\tau^2 = 0.00$, $I^2 = 0.00\%$, $H^2 = 1.00$

Test of $\theta_i = \theta_j$: Q(1) = 0.16, p = 0.69

Test of $\theta = 0$: z = -2.88, p = 0.00

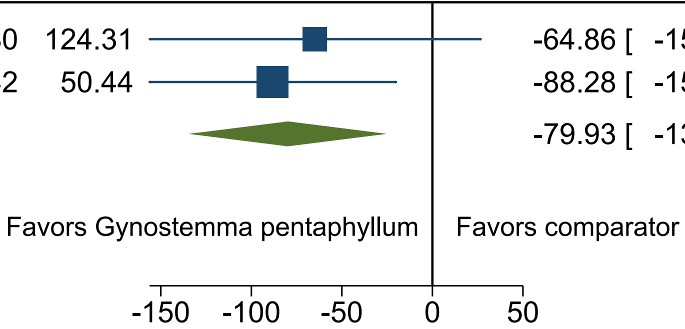

Random-effects REML model
